# Combining cognitive stimulation therapy and fall prevention exercise (CogEx) in older adults with mild to moderate dementia: a feasibility randomised controlled trial

**DOI:** 10.1186/s40814-020-00646-6

**Published:** 2020-07-25

**Authors:** Elizabeth Binns, Ngaire Kerse, Kathy Peri, Gary Cheung, Denise Taylor

**Affiliations:** 1 Physiotherapy Department, AUT University, Auckland, New Zealand; 2 Health and Rehabilitation Institute, AUT University, Auckland, New Zealand; 3 School of Population Health, University of Auckland, Auckland, New Zealand; 4 School of Nursing, University of Auckland, Auckland, New Zealand; 5 Department of Psychological Medicine, University of Auckland, Auckland, New Zealand

**Keywords:** Dementia, Cognitive stimulation therapy, Exercise

## Abstract

**Background:**

People living with dementia (PLwD) have a high fall risk as cognitive impairment compromises control of gait and balance. Fall prevention exercises that are effective in healthy older adults may not work for PLwD. Cognitive stimulation therapy (CST) has been shown to improve global cognition in PLwD. A programme which combines cognitive (CST) with physical exercises may reduce falls in PLwD. The aim of this study was to assess the feasibility of undertaking a full scale randomised controlled trial to test the effectiveness of CogEx in decreasing falls in PLwD. Specific objectives included recruitment strategy, data collection, outcome measures, intervention fidelity and facilitator/participant experience.

**Methods:**

A mixed methods feasibility randomised controlled trial recruited people from residential aged care. Inclusion criteria were ≥ 65 years old, Montreal Cognitive Assessment (MoCA) score of 10 to 26 and able to participate in a group. Participants were randomised to CST or CST combined with strength and balance exercises (CogEx). Both CST and CogEx groups were for an hour twice a week for 7 weeks. Descriptive statistics were used to report pre- and post-intervention outcome measures (MoCA, Geriatric Depression Scale–15, Quality of Life-Alzheimer’s Disease, Alzheimer’s Disease Assessment Scale—Cognitive 11, Brief Balance Evaluation Systems Test and Short Form Physical Performance Battery) and attendance. Qualitative analysis of participant focus groups and facilitator interviews used a conventional approach. Sessions were video recorded and exercise completion documented.

**Results:**

Thirty-six residents were screened with 23 participants randomised to intervention (CogEx, *n* = 10) or control (CST, *n* = 13). The assessments took 45 min to 1.5 h, and there was repetition between two cognitive measures. Ten facilitators completed training with the manualised programme. Exercises were combined into the hour-long CST session; however, limited balance training occurred with participants exercising predominantly in sitting. The facilitators felt the participants engaged more and were safer in sitting.

**Conclusions:**

The results demonstrated that while fall prevention exercises could be scheduled into the CST structure, the fidelity of the combined programme was poor. Other components of the study design need further consideration before evaluation using a randomised controlled trial is feasible.

**Trial registration:**

anzctr.org.au (ACTRN12616000751471) 8 Jun 2016, Australian New Zealand Clinical Trials Registry.

## Introduction

Dementia is a neurodegenerative disorder in which there is deterioration in memory, thinking, behaviour and the ability to perform everyday activities [1]. It mainly affects older adults, although it is not considered a normal part of ageing [1]. Globally, it is estimated there are 47.5 million people living with dementia (PLwD) and 7.7 million new cases diagnosed every year. It is predicted that the worldwide number of PLwD is likely to rise to over 75.6 million by 2030, and almost triple to 135.5 million by 2050 [2].

Cognitive stimulation therapy (CST) is a treatment developed for people with mild to moderate dementia. CST aims to enhance cognitive and social functioning using group therapy incorporating reality orientation, reminiscing, socialising and actively stimulating PLwD, while providing an optimal learning environment and the social benefits of a group. The therapy is standardised into a published manual [3] and guides the CST facilitator through set topics of engagement while allowing the group participants to choose the specific content. The 7-week programme is comprised of a 1-h group activity session twice a week. CST is the only evidence-based treatment recommended for people with mild to moderate dementia in the NICE dementia guidelines [4] based on the evidence that it can improve cognition in people with mild to moderate dementia over and above any medication effects [5, 6]. The Dementia Care Framework [7] in Aotearoa New Zealand recommends CST as one of only two specific treatments considered as *good practice* for PLwD. To support this recommendation, 1-day CST programme training of group facilitators occurred nationally [8], and CST is now available through community-based (CB) programmes and in residential aged care (RAC) facilities.

The incident rate for falls in community-dwelling older adults is 0.65 falls per person-year and for older adults living in RAC this increases to 1.7 falls per person-year [9]. Dementia is an independent risk factor for falls, and PLwD are twice as likely to fall and sustain an injury than those without dementia [10]. With the rise in the prevalence of dementia, falls in older adults with dementia are an area of serious concern in health care.

Falls in PLwD are multi-factorial [11], as they are in older adults [12]. Superimposed on the risk factors for falls in older adults are specific clinical features in PLwD that contribute to falls, the two most commonly recognised being cognitive impairments and gait abnormalities [13]. Cognitive impairments and gait abnormalities have been found to be interrelated [14] reflecting that gait is no longer considered a purely motor task but an activity that requires interaction with the environment, attention and executive function [13, 15, 16]. In PLwD, these abnormalities can be observed as impaired judgement, decreased walking ability, lack of visual-spatial perception and a loss of ability to recognise and avoid hazards [17].

Strengthening and balance exercises are the critical components in fall prevention interventions for community-dwelling older adults [18] and are highly associated with falls [19]. However, fall prevention interventions that work in healthy older adults may not work in PLwD. A meta-analysis identified only three randomised controlled trials assessing the effectiveness of exercise programmes to reduce falls in community-dwelling older adults with dementia. While all three studies included strength and balance exercises and the results looked promising, more research is required to ascertain these are important components to include in fall prevention interventions for PLwD [17].

This leads us to ask whether effective fall prevention for PLwD can be developed that incorporates physical and cognitive exercise? Is it possible to reduce falls risk by improving cognition with cognitive exercise as well as strengthening lower limbs and retraining balance in PLwD using a top-down (cognitive) and a bottom-up (physical) approach? CST is a programme that can improve global cognition in PLwD over and above the effect of medication and is available through a widespread, trained workforce in Aotearoa New Zealand. The highly structured CST programme lends itself to easily incorporating physical exercise without becoming complex, thereby using the existing workforce and not increasing the use of health resource to deliver fall prevention to this at-risk population. We hypothesise that combing fall prevention exercises with CST (CogEx) can deliver fall prevention to PLwD through already established dementia care providers nationally. Prior to embarking on a full-scale clinical trial, there are several feasibility issues to address.

### Aim

The aim of this study was to assess the feasibility of undertaking a full-scale randomised controlled trial to test the effectiveness of CogEx in decreasing falls in PLwD. For the full-scale RCT, the primary outcome is falls. This study will explore which secondary outcome measures are appropriate to measure the other potential benefits of CogEx and add important information for statistical modelling in the future RCT.

The specific objectives were as follows:

1. To test the recruitment strategy of facilities and individuals, percentage recruited and the resultant characteristics of PLwD who participated
2. To test the appropriateness of data collection procedures and select secondary outcome measures
3. To test combining fall prevention exercise into CST
4. To test training of CST facilitators to deliver CogEx
5. To test intervention fidelity of CogEx delivered by facilitators
6. To explore the facilitators’ perceptions of delivering CogEx
7. To explore the participants’ experience of CogEx

Establishing the feasibility of collecting fall data was not a study objective. The research team has a feasible and accurate method of recording falls which involves blinded assessors auditing the incident reports at an RAC facility for falls over a specified date range. We are currently using this method in another fall prevention study being conducted in RAC facilities [20].

## Methods

### Design

A mixed methods study design was used to evaluate the feasibility issues in two settings (Residential Aged Care and Community Based) [21]. This included quantitative outcome measures in a randomised controlled study comparing CST (control group) with CogEx (intervention group) and qualitative evaluation of the experience of study participants, group facilitators and blinded assessors. Ethical approval was given from the New Zealand Health and Disability Ethics Committee (16/NTB/121).

### Setting

The Residential Aged Care (RAC) facility had a mixture of residents living in the rest home and private hospital-level care. For the community-based (CB) setting, a regional non-government organisation (NGO) supporting PLwD and their family delivering CST in the community had agreed to take part.

### Recruitment of individual participants

The initial inclusion and exclusion criteria for participants are listed in Table 1.

**Table 1:** Eligibility criteria

Inclusion criteria	1. Aged 65 years or older
	2. Living in their own home or in residential aged care
	3. A diagnosis of mild to moderate dementia
	4. Mobile with or without an assistive device
	5. A MoCA score between 15–26 out of 30
	6. Able to have a meaningful conversation
	7. Able to hear well enough to take part in small group discussion
	8. Able to see well enough to see pictures
	9. Likely to remain in a group for 1-h
Exclusion criteria	1. A recent significant medical illness
	2. Unable to participate due to severe visual or hearing impairment
	3. Wheelchair/bed bound or unable to walk
	4. Receiving terminal or palliative care.

*MoCA* Montreal Cognitive Assessment (0–30, a higher score = better cognition)

The RAC clinical manager used their knowledge of the residents and applied the inclusion and exclusion criteria to screen all residents and identify those who may be eligible to participate in the study. The clinical manager then explained the research to individual residents and invited them to participate as well as contacting the Next of Kin (NoK) to explain the research and their relative’s potential involvement. If agreeable to participating in the study, the participant and their NoK’s names and contact details were forwarded to the primary researcher who then gave resident contact details to a research assistant (RA).

Potential CB participants and their NoK were informed about the study by an NGO keyworker and given the study information. The keyworker followed-up by phone a week later. If agreeable to participating in the study, the participant and their NoK‘s names and contact details were forwarded to the primary researcher who then gave the potential participant's contact details to a research assistant (RA).

Participants were asked to give verbal and written consent by the RA before completing the baseline assessments, and the NoK were asked to give verbal or written assent by the clinical manager or the primary researcher.

### Procedures

The participants were enrolled by the RA prior to baseline assessment. At baseline assessment demographic information, medical conditions including a history of falls, current medication and use of walking devices were recorded for each participant as well as asking if they had experienced pain or fatigue over the past few days [22]. Outcome measures were also completed. The RA assessors were blinded and remained blinded throughout the study. It was not possible to blind the staff facilitating the groups or participants to group allocation.

### Randomisation and allocation

The primary researcher created a computer-generated sequence and used this to randomise participants. The primary researcher allocated participants to a group within the setting where they lived and then sent the group lists to the staff who were facilitating the groups.

As this was a feasibility study, no sample size was statistically calculated. The intended sample size of 32 participants was based pragmatically on the usual size of a CST group being 8–10 participants and the feasibility study using two CogEx groups and two CST groups.

### Interventions

The group facilitators were staff who already ran group exercises and activities, had completed the 1-day CST training and were asked by their manager to facilitate the groups for the study. None of the facilitators had run CST prior to the study. The facilitators completed CogEx training in small groups. The training sessions were an hour long and included working through the CogEx manual (available on request), discussing the principles of the programme (safety, fun, session activities, challenge, effort, 30 s bouts of exercise, safety), practicing the 3-min exercises to a song, practicing the strength and balance exercises and the progressions, discussing the attendance and exercise record sheet and practicing with the 30 s interval timer. The facilitators were asked to check with participants at the start of each session if they had any pain. The TIDiER Checklist is an additional file (see Additional file 1).

### Cognitive stimulation therapy (control group)

CST was delivered as per the CST manual for group leaders [3] which was a group session for 1 h twice a week for 7 weeks, with each of the 14 sessions having a different theme.

### CogEx (intervention group)

CogEx was CST (as above) with aerobic and progressive strength and balance exercises embedded. As socialisation and engagement are key elements of CST, it was important to keep both groups the same length of time; otherwise, if CogEx was longer, those participants would receive longer contact time and therefore more socialisation and engagement. To keep the CogEx sessions the same length of time as CST, 3 min of aerobic exercise was included during the welcome and the farewell song (6 min in total) and 10 min of strengthening and balance exercises occurred in the body of the session.

The aerobic exercises were continuous for 3 min and included 30 s bouts of exercises in standing (walking on the spot, stepping side to side, washing machines, turning to tap your neighbour, washing machines repeated). The strength and balance exercises were informed by clinical experience, previous fall prevention research [20, 23] and an understanding of the physiological systems of balance and rehabilitation principles. This included the following:

- *Motor control*—muscles are turned on according to the task [24, 25] and underpins specificity of exercises; therefore, exercises were functional, i.e., in weight-bearing and incorporated everyday movement.
- *Overload—*the body responds to a stimulus over and above what it normally experiences [26] so to improve balance it must be challenged.
- *Balance is multisystem* exercises—included muscle strengthening, vestibular adaptation and balance strategy retraining.

The exercises targeted major muscle groups, range of motion, physiological systems and were designed to be in weight-bearing (see Additional file 2). The exception was the vestibular ocular reflex exercises which were in sitting. Strengthening exercises were chosen that incorporated balance for example standing and flexing the knee to bring the heel toward the bottom strengthens hamstrings but also decreases the base of support with standing on one leg. Each exercise was completed for a period of 30 s.

The exercises were the same at each session so that the repetition might result in participants becoming familiar with the exercises and their balance self-efficacy increasing. Exercises were progressed individually according to the participant's ability by decreasing hand support and encouraging more repetitions within the 30 s.

CogEx was manualised and included the programme principles, session structure, photographs of exercises with instructions and progressions and a one-page exercise sheet for each session that could be used to plan and record the exercises (see Additional file 3).

All sessions (CST and CogEx) were run by two facilitators as per the usual CST practice [27]. The 1 h sessions were on days and times decided by the RAC and NGO.

### Data collection

Quantitative and qualitative methods were used to answer the study objectives.

### Outcome measures

Outcome measures were chosen that were widely used with older adults or PLwD; however, it was necessary to assess the appropriateness of the measures for this particular population of older adults as well as how long it took to complete all the outcome measures (study objectives 1 and 2). The MoCA score (to assess cognition) was completed first to screen participants for study eligibility [28]. Then, depression assessed with the 15-item Geriatric Depression Scale (GDS) [29], quality of life with the Quality of Life-Alzheimer’s Disease (QOL-AD)—Version for the person with dementia [30], cognition with Alzheimer’s Disease Assessment Scale—Cognitive (ADAS-Cog11) [31], balance with the Brief Balance Evaluation Systems Test (Brief BESTest) [32] and functional mobility with the Short Physical Performance Battery (SPPB) [33].

An attendance sheet documented participants’ attendance at each session, and a record sheet of each CogEx session was kept documenting the level of the strength and balance exercises completed (study objectives 3 and 4) (see Additional file 3).

Adverse events were monitored by the facilitator asking each resident at the start of each session how they were. All outcome measures were reassessed the week following completion of the 7-week programme.

### Qualitative evaluation

Focus groups were used to generate information about participants’ experiences of CogEx sessions; they were facilitated by the primary researcher immediately after the final CogEx session (study objective 7). The participants were asked what they liked or did not like about the sessions, what they thought about the exercises and any changes they would like to see. The focus groups were audio-recorded and transcribed verbatim.

Semi-structured interviews were used to generate information about the facilitator’s experience of running the groups (study objective 6). The primary researcher interviewed each facilitator after completion of the last group session. The interview was at a time that suited the facilitator and sought to ascertain their thoughts about the programme, the structure, the length of the session and the exercises. The interviews were audio-recorded and transcribed verbatim.

Semi-structured interviews were used to generate information about the assessor’s experience of assessing the participants (study objective 2). The primary researcher interviewed each assessor after completion of the post-intervention assessments. The interview was at a time that suited each assessor and sought to ascertain their thoughts about using the outcome measures with the participants and how long each assessment took. The interviews were audio-recorded and transcribed verbatim.

A video camera was set up by a facilitator for each session. The camera was on a tripod in the corner of the room and recorded participant interactions and engagement (CST and CogEx) as well as how participants transitioned from one task to the other.

### Data analysis

Descriptive statistics were used to describe the number of residents recruited (percentage), the group demographics at baseline, pre- and post-intervention outcome measures (means, standard deviations), change in outcome measures (difference, 95% confidence intervals) (objectives 1 and 2), session attendance (percentage) (objective 5) and exercise session content (minutes in sitting and standing) (objectives 3–5).

Qualitative analysis of the focus groups and semi-structured interviews (facilitators and assessors) used a conventional approach to content analysis informed by Hsieh and Shannon [34]. Common ideas were identified within and across the transcripts, then grouped into themes. The qualitative data were used to examine and the assessor’s perception of the data collection (objective 2), identify acceptability of the session to the participants and examine facilitator perception of session delivery (objectives 6 and 7). The video of sessions was viewed to observe how the participants engaged with the exercises and transitioned from one task to another.

## Results

There were changes to the original protocol after the commencement of the study.

### Setting

The research groups first ran at the RAC. When the NGO went to identify CST groups for the trial, they discovered they were unable to provide any groups in the required timeframe. They found that three CST groups per geographical area per year met their current demand. No groups were scheduled to start until after the study funding had expired. Therefore, a second RAC facility (RAC2) was recruited to participate in the study. RAC2 was a facility providing a mix of assisted living, rest home and private hospital-level care.

### Recruitment of individual participants

Changes in the study settings resulted in alterations to some of the initial eligibility criteria (Table 1):

- Inclusion criteria 2: Due to the NGO no longer taking part in the study, this was modified to “living in residential aged care”.
- Inclusion criteria 3: Staff at RAC1 explained that if cognitive decline developed after admission to RAC, a resident may not be formally diagnosed with dementia. On this information, inclusion criteria were expanded to include “staff identified cognitive problems”. In RAC2, staff only knew an assisted living (apartment with nursing services provided) residents’ diagnosis if the person chose to share their medical history with care staff. Therefore, the study was advertised as seeking people to participate who felt they may have memory problems as well as inviting people with mild to moderate dementia or staff identified cognitive problems. Inclusion criteria and information sheets for participants and families were modified to include “self-identified memory problems”.
- Inclusion criteria 5: The eligibility cut off score of > 15/30 on the MoCA had been used previously by the research team members in community-based CST research. However, of the first four residents assessed in RAC1, only one had a MoCA > 15/30. In a discussion with the research team, the cutoff MoCA score was lowered to > 10/30 to screen residents as eligible to participate in the study.

Recruitment at RAC2 involved the clinical manager inviting residents, as well as the research being advertised in the village flyer and a talk and information sheets given to interested residents.

The trial concluded as planned with all groups completing their 7-week programme and post group assessments. No adverse events were reported throughout the trial.

**Fig. 1:**
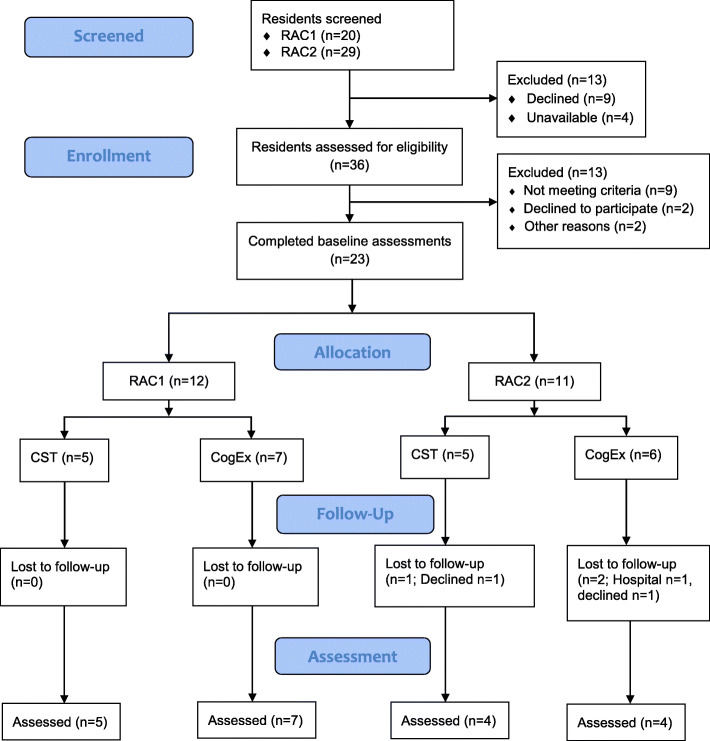
Flow of participants through the trial

The flow of participants through the study can be seen in Fig. 1. At RAC1 recruitment from 94 residents occurred from January to March 2018. Of the 20 residents identified by the clinical manager as appropriate to participate in the study, 19 volunteered to participate and of those 12 were eligible. The recruitment for RAC1 was 13% (12/94); of those who were invited and eligible, all took part (100%). At RAC2 recruitment from 52 residents occurred from May to July 2018. Of the 29 residents self-identified or identified by the clinical manager as appropriate to participate in the study, 17 volunteered to participate and of those 11 were eligible. The recruitment rate for RAC1 was 21% (11/52). Of those who were invited and eligible, all took part (100%).

Of the nine residents who did not meet the inclusion criteria, three had a MoCA score > 26/30, four had a MoCA score < 10/30, one was awaiting a lower limb prosthetic and unable to stand, one was visually impaired and unable to do the pen and paper tests; the other reasons were one resident became distressed during the assessment, so it was stopped and one resident could not stay on topic long enough to complete any of the tests.

No participants were lost to follow-up at RAC1, and three were lost to follow-up RAC2 (one was in hospital, one had stopped going to sessions and declined reassessment, one was emotionally unwell, could not complete the MoCA and the assessment was stopped). Of those lost to follow-up, one was in the CST group and two were in the CogEx group.

### Randomisation and allocation

At RAC1, CST ran first followed by the CogEx group. This was a pragmatic decision by management due to staff availability and allowed the facilitators to experience running a CST group before running CogEx. The research team was concerned that if all participants completed baseline assessments in the same week that those waiting 7 weeks for their group to start might deteriorate. For this reason, once residents and their NoK agreed to participate in the study, they were randomised and then assessed in the week prior to their group starting.

At RAC2, CST and CogEx groups were run in parallel; therefore, participants were randomised following baseline assessment.

### Quantitative evaluation

#### Participants

The baseline group demographics in Table 2 demonstrate that the groups were similar except for the MoCA, ADAS-Cog 11 and mobility. On the MoCA, the CogEx group lower mean of 16.0 (SD 4.2) was in the moderate cognitive impairment band of 10–17 while the CST mean of 18.0 (SD 5.6) was in the mild cognitive impairment band [35]; however, the 2-point difference is smaller than the 4-point minimum detectable change to be sure the difference is not due to measurement error [36]. Similarly, on the ADAS-Cog 11, the CogEx group had a lower mean ADAS-Cog 11 score of 17.4 (SD 5.9) compared to the CST mean of 15.0 (SD 10.4); however, a definitive cutoff score for dementia has not been established. If a score of ≥ 17 for dementia [37] is used then the CogEx group is classed as having dementia but if > 18 [38] is used, then both groups have a worse score than 5 being normal [39] but not classified as dementia. More participants in CogEx used an assistive device when walking.

**Table 2:** Baseline group characteristics and outcome measures at baseline and reassessment

	Baseline		Reassessment	
	CST	CogEx	CST	CogEx
Number of residents randomised	10	13	9	11
Mean age in years, (range)	83.6 (71-95)	87.5 (81-95)	–	–
Female, *n* (%)	8 (80)	9 (69)	–	–
Uses a walking device, *n* (%)	4 (40)	9 (69)	–	–
Medication, mean (SD)	5.2 (2.5)	6.8 (3.6)	–	–
MoCA, mean (SD)	18.0 (5.6)	16.0 (4.2)	18.3 (6.9)	14.6 (3.3)
GDS-15, mean (SD)	3.8 (3.4)	2.9 (2.8)	4.3 (5.1)	2.6 (2.0)
QoL: AD, mean (SD)	37.7 (5.8)	37.2 (6.4)	37.2 (5.7)	37.6 (4.8)
ADAS-Cog 11, mean (SD)	15.0 (10.4)	17.4 (5.9)	14.9 (9.5)	18.4 (5.5)
Brief BESTest, mean (SD)	8.5 (5.5)	9.0 (6.0)	10.3 (5.2)	8.2 (5.1)
SPPB				
Balance score, mean (SD)	2.4 (1.4)	2.4 (1.6)	3.0 (1.0)	2.3 (1.3)
Gait score, mean (SD)	2.3 (1.4)	2.6 (1.2)	2.0 (1.0)	2.2 (1.3)
Chair stand score, mean (SD)	0.8 (0.4)	1.3 (1.3)	1.2 (1.1)	0.9 (0.9)
Total score, mean (SD)	5.5 (2.1)	6.3 (3.8)	6.2 (2.2)	5.4 (2.9)

*MoCA* Montreal Cognitive Assessment (0–30, a higher score = better cognition); *GDS-15* Geriatric Depression Scale—15 (0–15, A score of > 5 = likely to have depression); *QoL* AD quality of life: Alzheimer’s disease (13–52, a higher the score = better the quality of life); *ADAS-Cog 11* Alzheimer’s Disease Assessment Scale—Cognitive 11 (0–70, a lower score = better cognition); *Brief BESTest* Brief Balance Evaluation Systems Test (0–30, a higher score = better balance performance); *SPPB* Short Form Physical Performance Battery (item score 0–4, total score 0–12, a higher score indicates better performance)

All participants were reassessed in the week following the last session (Table 2). For RAC1, CST post group assessments occurred 14–18 May 2018 and CogEx 3–7 July 2018; and for RAC2, both group post group assessments occurred 29—30 August 2018.

The difference in group baseline and reassessments measures in Table 3 illustrate that no clinically meaningful change was observed in any outcome measure for either group.

**Table 3:** Change in group outcome measures after the intervention (with 95% confidence intervals)

	CST *n* = 9	CogEx *n* = 11
MoCA	1.2 (− 1.0, 3.5)	− 0.6 (− 2.2, 0.9)
GDS-15	0.1 (− 2.2, 2.5)	− 0.3 (− 1.8, 1.3)
QoL: AD	0 (− 2.4, 2.4)	0.5 (− 2.1, 3.0)
ADAS-Cog 11	− 1.1 (− 6.7, 4.6)	− 0.3 (− 2.7, 2.2)
Brief BESTest	1.3 (− 1.5, 4.2)	1.0 (− 1.1, 3.1)
SPPB		
Balance score	0.4 (− 1.0, 1.8)	0.1 (− 0.5, 0.7)
Gait score	− 0.3 (− 1.4, 0.7)	− 0.2 (− 0.6, 0.2)
Chair stand score	0.4 (− 0.2, 1.1)	− 0.1 (− 0.6, 0.4)
Total score	0.6 (− 0.9, 2.0)	− 0.2 (− 1.0, 0.6)

*MoCA* Montreal Cognitive Assessment (positive difference = improvement); *GDS-15* Geriatric Depression Scale–15 (a negative difference = improvement); *QoL* AD quality of life: Alzheimer’s disease (a positive difference = improvement); *ADAS-Cog 11* Alzheimer’s Disease Assessment Scale—Cognitive 11 (a negative difference = improvement); *Brief BESTest* Brief Balance Evaluation Systems Test (a positive difference = improvement); *SPPB* Short Form Physical Performance Battery (a positive difference = improvement)

Additional files show the individual participant change scores by group (see Additional files 4 and 5).

### Interventions

Ten facilitators trained to deliver CogEx (RAC1 *n* = 5, RAC2 *n* = 5). RAC1 CST was delivered 26 March–9 May 2018 and CogEx 14 May–29 June 2018; RAC2 CST and CogEx were delivered 16 July–29 August 2018. Three of the four group sessions were held in lounges or activity rooms with participants sat at tables except for RAC2 CST where the group sat in a semi-circle in lounge chairs.

### Attendance

RAC1 kept attendance sheets for all CST and CogEx sessions. RAC2 kept attendance sheets for 12/14 CogEx sessions and no CST sessions. The percentage of attendance was calculated as the number of participants attending the total number of available group sessions; participant attendance rate per session was also calculated. CST (*n* = 5) average session attendance was 92% (80–100%) and CogEx (*n* = 13) attendance rate was 55% (17–100%).

### Class record sheets

The CogEx facilitators recorded the level of strength and balance exercises performed in the 10 min block; the 3 min of aerobic exercise (continuous movement) during the welcome and the farewell song was not recorded as these exercises were set and did not change over the 7 weeks of the programme. The RAC1 facilitators kept and returned the record sheets, the RAC2 facilitators did not. Exercises were performed in bouts of 30 s. Table 4 illustrates the time spent and number of exercises completed in standing at each session. Not all participants performed calf raises in standing. Sit to stand and calf raises were two of the five exercises performed at the start and end of the 10 min block; therefore, each bout of an exercise was counted individually.

**Table 4:** RAC1 CogEx standing exercises completed at each session

Week	Session	Standing exercises (*n*/16)	Total time in standing (minutes)
1	1	2	1
	2	2	1
2	3	Not recorded on a sheet	–
	4	Not recorded on a sheet	–
3	5	2	1
	6	2	1
4	7	Public holiday No session	–
	8	2	1
5	9	2	1
	10	2	1
6	11	4	2
	12	2	1
7	13	2	1
	14	2	1

### Qualitative evaluation

Only the CogEx qualitative results are presented here to answer study objectives 6 and 7.

### Participant focus groups

Both facilities had a culture of resident inclusiveness which meant a resident could take part in any activity that was on offer if they wanted to. Therefore, residents who were not research participants attended the sessions. This inclusiveness resulted in everyone at the last CogEx sessions being invited to participate in the focus group; they were shown the recorder and it was explained that what they said would be recorded, transcribed but not with names and this would be used for research purposes only. They were given the opportunity to leave however, everyone stayed and this resulted in a mix of participants and non-research participant residents take part in the RAC1 and RAC2 focus groups. It was not possible to remove non-research participant comments from the analysis as transcriptions were anonymised. The ethics committee who had approved the study were notified of the protocol violation and gave further approval.

Seven residents took part in the RAC1 focus group (participants *n* = 4 and non-research participant residents *n* = 3) and six residents in the RAC2 focus group. At RAC2, the mix of RAC2 participants and non-research participants was unknown as there was no attendance sheet for the final CogEx session. The answers given by RAC1 participants tended to be briefer than those given by RAC2 participants.

The two main topics discussed at the focus groups were the group and the exercises.

Overall, the participants enjoyed being a part of the group, what they did in the sessions and wanted the sessions to continue. They enjoyed doing the sessions together and took pleasure in that. They experienced a feeling of being known and knowing as a result of the discussion topics that were used in the CST structure:

Just the fact that somebody knows that you are (name)…all of us in a way have got to know each other better (female, RAC2)

…we have the questions, we play the games whatever and it means you can beat (others)…that it’s not just sitting here nattering to another bunch of old people (female, RAC2)

The participant’s comments on the exercises were mixed. There was also discussion at RAC2 of using music to make exercise more enjoyable:

Oh, that was quite good, gets the whole body moving (male, RAC2)

I’ve been doing them in my chair and quite happy (female, RAC2)

I would have liked to have been able to do them better (male, RAC1)

I remember not being able to do some of them, balancing and things. Some of them were very hard (female, RAC2)

One participant declined reassessment as she had stopped attending after session 2 or 3. She was not part of the focus group but wanted her thoughts known as she found the sessions did not make sense, did not help and did not find the topics enjoyable.

### Semi-structured interviews

The RAC1 CogEx facilitators were invited to be interviewed and two accepted (*n* = 2/5). They were interviewed individually at the facility at a time that suited them following completion of CogEx. The RAC2 Manager offered to talk about CogEx and asked for the facilitators not to be interviewed as the exercise component of CogEx had been discontinued from the sessions in week 2 or 3.

### Facilitators

The main topics of the facilitators’ comments were time, engagement, improvement and exercises.

#### Time

Facilitators combined exercises into the welcome and farewell group song and used the 30 s interval timer for each exercise as planned. To keep the sessions to an hour long and the same length as CST, the facilitators removed the newspaper reading component of CST. They felt that not everyone could see the newspaper to read it, not everyone had an opinion on the news and the group was split when only a few participants gave their opinion whereas the exercises were all inclusive.

#### Engagement

The facilitators described participants engaging more as the weeks progressed, with participants enjoying singing along with the songs and doing the movements:

They start (singing) “It’s a long way to Tipperary” so you know they are really enjoying it, I think it’s great, even the heads are going from left to right, nobody’s complaining this is sore (Facilitator 1)

One lady in the wheelchair was unable to try but her feet were moving so she was aware that something should be happening…engaging her feet (Facilitator 2)

The facilitators also felt that when participants physically touched each other during a trunk rotation exercise (when they reached their arm across their body to tap their neighbour on the shoulder), this increased their engagement with others in the group:

…they touch and they said o that is nice you gave me your hand (Facilitator 2)

I noticed one or two of them who weren’t quite sure what they were doing, the other residents actually engaged with them (Facilitator 2)

There was also a sense of teamwork with participants copying each other and helping each other out during the group activities and exercises:

Other residents helping those who were slightly hesitant to stand up (Facilitator 2)

#### Improvement

The facilitators described that as the weeks progressed, the participants became more familiar with the exercises, required less prompting and remembered that the session started with a song and movement. The facilitators could see the participants getting better at the exercises:

…they are getting even lower you know bending…they have been here twice a week touching the ground, they are really touching the ground (Facilitator 1)

…some of them now are standing up (Facilitator 1)

One lady in particular made full use of her 30 seconds whereas another couple of people would just be able to do one or two (Facilitator 2)

Most of the sitting ones are very good (Facilitator 1)

#### Exercises

The facilitators described doing almost all the exercises in sitting. The two standing exercises that were performed were sit to stand and calf raises. A variety of reasons were given including the setup of the room, safety and it does not matter as long as participants moved:

I am scared that they will fall because they are standing up now…that’s fine and then they want to sit back so yeah go and sit back so the most sitting ones are very good (Facilitator 1)

Not many of them stood…most of them preferred to remain seated most of the time (Facilitator 2)

I am happy with the exercises, only the standing ones we didn’t really do that, maybe if we had another time only the standing ones, practice a lot on that you know, like sideways walking. Now we have seven but sometimes you have 11 and that’s too many then to look after especially because everybody is a bit frail (Facilitator 1)

…standing up and tip toes was fine… everything we do it in the chair…it doesn’t matter as long as they move (Facilitator 1)

The facilitator interviews description of performing almost all exercises in sitting was supported by video and the time spent standing in each session taken from the class record sheets (Table 4).

### Manager

The RAC2 manager recalled asking the facilitators how the groups were going and finding out that the exercises had been discontinued from CogEx in week 2 or 3. The manager described an aged care industry dynamic of staff not wanting someone to feel left out of an activity and so if one person could not do something, then whatever it was would be discontinued:

…she said well we have got a couple of people who can’t do them (the exercises) so didn’t think it was very fair on the others that they couldn’t do them…people do this all the time in aged care they feel sorry for the person who can’t do it so therefore nobody else can take part and that’s an interesting dynamic of this industry

The manager felt if there had been overall supervision of the groups by management that this may have kept the groups on track; however, it was not their role and maybe if they had a diversional therapist then, they would have overseen and been in charge of the project. Another suggestion was a visit by the researcher to check on the groups and if this had occurred the exercises could have been restarted:

…I think it would have been good to have someone with the research or someone just come back 2 or 3 weeks into it and just remind us how it’s going and just having a review just a couple of weeks into it…I think that would have been good (and) got them back on track very quickly

The manager described the difference in ages, personalities and experience of the staff who trained to be facilitators. Some staff were new to diversional or recreational therapy, so CST had been viewed as a structured way to train staff:

…I wanted them to do CST so that they don’t get into the traditional method of activities or social interaction but start to think about residents and their needs and to get the most out of them and that’s what the spin off for us has been that the other staff are very much more engaged

Other factors that the Manager felt affected the CogEx group was morning being a challenging time of day to run a group due to the staff being busy and if someone called in sick it was hard to find cover; that the small room CogEx was run in was harder for participants to move in and participants having to walk through a part of the facility where residents were in hospital-level care may have been off-putting for participants attending the CogEx group.

### Assessor semi-structured interviews

All assessors (*n* = 3) were interviewed individually at a time that suited them at the university. As this was a small number of people, repetitive comments around topics are presented rather than themes. The assessor’s main comments related to time, repetition and not feeling comfortable.

#### Time

The six outcome measures took between 45 and 90 min to complete. The assessors recalled the assessments took longer at RAC1 as the participants were cognitively slower, needed to be redirected to answer questions and motivated to continue. Assessments were often interrupted at RAC1 by staff giving medication or a cup of tea. The assessors recalled a few of RAC1 participants commenting on the number of questions they had to answer with some becoming bored, agitated or emotional when answering questions about marriage and family. The assessors felt that RAC2 participants were more independent and appeared to enjoy the challenge of the tests.

#### Repetition

The assessors identified repetition of items of the MoCA and ADAS-Cog 11 and described participants also being aware of this:

...they are familiar with the question and they might do better just like for example the drawing of the cube…several of them mentioned that “I have done this before” and it’s “I know I can’t do this, it is difficult for me and it’s there again” (Assessor 2)

The assessors felt that some participants looked a little confused being asked the same questions during the assessment such as the orientation questions of date, month, year, day and place.

#### Not feeling comfortable

The assessors felt uncomfortable with the level of challenge of some balance tests with all three assessors commenting on Brief BESTest items 5 and 6 that elicit a compensatory stepping strategy. They felt uncomfortable doing these tests and gave a variety of reasons such as the age of participants, the restricted space if assessing a participant in their room and not feeling it was very safe to do this test. One assessor also commented on testing one leg standing in the SPPB, saying that the participants didn’t like it:

A bit tricky like to stand on one leg, they would go “I don’t do that usually why would I do that, why are you testing something that I am not even trying to do?” (Assessor 3)

### Video

All the video footage was observed to analyse participants’ engagement and transitions from one activity to another. The video captured most participants however, as they were sat around a table less than half participants’ faces were visible. It was not possible to analyse facial expression or body language for the level of engagement at each session.

There were smooth transitions from one task to another such as from the welcome song to the exercises and the video supported the facilitators’ semi-structured interviews and documentation of doing almost all exercises in sitting.

## Discussion

The results of the CogEx feasibility study demonstrated that while fall prevention exercises can be incorporated into the CST schedule, the fidelity of the combined programme was poor and other components of the study design need further consideration before evaluation using an RCT would be feasible.

### Objective 1. To test recruitment strategy, percentage recruited and the resultant characteristics of PLwD who participated

An ideal recruitment rate was not set prior to the study. Recruitment of PLwD into research trials has been acknowledged as a problem in many countries [40], so the results from this study were to inform recruitment rates for a future study. When designing the study, the numbers of PLwD registered with the NGO was known but not the number of residents with dementia living in the RAC. Recruiting from the community was problematic due to the limited number of CST groups the NGO planned to run annually. Similarly, in RAC all potentially eligible residents were invited by the manager yet after screening less than the 16 were eligible at each facility. Lowering the MoCA score to that used in other CST studies [41] was appropriate for RAC and contributed to a higher number of residents being recruited; however, the percentage recruited at both RAC1 (13%) and RAC2 (21%) was low.

A fully powered fall prevention study requires hundreds of participants [42–44]. To recruit a large enough sample of participants for an RCT, all CST deliverers in Auckland (whether community-based or RAC) would need to be recruited or a multi-centre trial undertaken.

### Objective 2. To test the appropriateness of data collection procedures and select secondary outcome measures

The outcome measures used in this study were based on those in Spector et al.’s original pilot study and RCT [45, 46] with the addition of two balance and lower limb measures. However, the MoCA was used instead of the Mini-Mental Scale Evaluation (MMSE) to screen cognition firstly due to the MMSE no longer being freely available [47], and secondly, recent work has identified the MoCA as more sensitive to older adults with mild cognitive impairment [48, 49] and can measure change over time [50, 51]. The assessors and participants noted the repetition between the MoCA and the constructional praxis and orientation sections of the ADAS-Cog-11 and this may have impacted on some participant’s performance and agitation with the tests. The MoCA took less time than the ADAS-Cog 11 to administer and needed only the paper copy of the test and a pen while the ADAS-Cog 11 required a large kit of equipment. The MoCA also has banded scores to describe a person’s level of cognitive impairment [35] while the ADAS-Cog 11 has a normal score established for healthy older adults but a definitive cutoff score for dementia is still being debated as is what constitutes a meaningful clinical change [52]. Of these two tests, the MoCA is currently the better choice to ascertain a person’s level of cognitive impairment for use in future studies unless more detail was required as to the type of cognition being impacted.

The outcome measures did not show change after taking part in CogEx; however, given that only 1 min of exercise in standing was completed this could be expected. The number of participants was also too small for inferential analysis or statistical modelling to be undertaken.

The Brief BESTest was chosen to assess balance as it assesses six subcategories that contribute to the maintenance of balance [32] and therefore provides more insight as to why balance is decreased; this version is also the quickest to complete of all BESTest versions [53]. The assessors felt uncomfortable with the more challenging balance tests (reactive postural response) despite having trained to do the outcome measures and practicing them on each other. The assessors also felt uncomfortable assessing the one-leg stand component of the SPPB because of some participants’ reaction to being asked to do something they said they never do.

Assessing balance in frail, older adults who perform fewer incidental activities of daily living in RAC is challenging, as in that environment the population is not homogeneous. Our knowledge of physical tests has not kept up with the global population increase of older adults. There is a lack of normative values in outcome measures for older adults living in RAC as most work to date has been on healthy older adults living in the community [54]. An example of this is of the outcome measures used in this study only three have had a minimal detectable change identified: MoCA 4 points [36]; Brief BESTest 4 points [55]; SPPB total 1 point [56]; however, only the Brief BESTest established this for a population of older adults living in RAC. Consideration should be given to this when selecting measures for future studies.

The battery of assessments took too long and some participants became fatigued or agitated. With the growing evidence of the benefit of CST on cognition and quality of life [6], the number of tests could be reduced to focus purely on changes in physical performance in response to CogEx.

The class record and attendance sheets captured the desired information however asking the facilitators to return them weekly would have given the research team oversight of the level of exercises being delivered and created the opportunity to discuss with the facilitators their choices and possibly intervene.

The focus groups were challenging due to research non-participants taking part. The RAC facilities were offering CST as part of their resident activity programme and agreed for the groups to be used for research. As the RACs hosted the research they retained control of the groups and applied their inclusiveness ethos, which resulted in non-research participants taking part in the groups. This also created an ethical issue as these residents had not given written informed consent.

Focus groups were held immediately after the last session to accommodate for cognitive impairment so that participants knew what group activity and exercises the questions related to. Before the focus groups took place, the researcher explained what questions were going to be asked and why, the use of the voice recorder was explained and the opportunity to leave and not take part was given. Focus groups had been chosen when designing the study in order to generate discussion between participants however some focus group participants had poor hearing, and this made their participation in the focus group challenging. The focus group transcription was anonymised making removal of non-research participant comments impossible. Individual interviews with research participants would address the above problems but remove the creation of information that occurs when participants talk between each other.

### Objective 3. To test combining fall prevention exercise into CST

The RAC1 facilitators successfully incorporated the additional exercises and kept the CogEx sessions the same length as CST (1 h). They achieved this by firstly, removing the discussion of topics from the newspaper as they found the group did not engage with this. And secondly, by socialising with morning tea after the session rather than as a form of welcome to the group as CST in the community does. In RAC, morning tea is a part of the daily routine so having morning tea after the session helped CogEx to run within the RAC normal schedule.

### Objective 4. To test training of CST facilitators to deliver CogEx

The additional exercise time was scheduled into the CST structure; however, almost no standing exercises were performed. This could be due to CogEx facilitator training being insufficient. Training was a 1 h session working through the manual, discussing the principles of the programme, practicing the different levels of the exercises and practicing using the timer. This pragmatic approach had been used by the research team previously [57] and was how implementation was envisaged in order to prevent training costs being a barrier. Removing any picture or option of sitting exercises from the manual could act to prompt the facilitators to focus on the importance of exercising in standing to improve the participants’ balance. One facilitator’s comment that it did not matter what the participants did as long as they were moving suggests that the difference between fall prevention exercise and activity was not understood.

The lack of standing exercise could also be due to the industry dynamic referred to by the manager of making sure everyone does the same thing, alluding to accepted cultural norms within a facility. It could be that this group of health care workers are very well trained to err on the side of safety and comfort versus physiotherapists who are trained to encourage people to work to their limits and know challenging balance is important in order to stimulate physiological change. The exercise programme while appearing straightforward requires a skill set that is not an inherent quality of health care workers in RAC who routinely lead activities with large groups of residents in sitting.

Ongoing training such as a physiotherapist attending one session a week to support the facilitator may have helped to grow the confidence and understanding of the facilitators in encouraging participants to stand and progressing the exercises. Increasing the time the physiotherapist is involved with delivering CogEx training and implementation also increases the implementation costs of the programme, but this would be beneficial if they facilitators gained confidence, knowledge and long-term skills to deliver future CogEx programmes independently.

### Objective 5. To test intervention fidelity of CogEx delivered by facilitators

This study contained measures of delivery and engagement as categorised by Walton, Spector, Tombor and Richie that when triangulated provided a description of the fidelity of the intervention [58]. Fidelity measures of delivery were the class record sheets (session content) and the facilitator and manager semi-structured interviews, all which illustrated that standing exercise content was minimal. While the video of sessions was recorded, ethical approval was for the video to be used to observe participant engagement and transition from one task to another, not to observe the content of the session or the facilitators. The active ingredient of the intervention (weight-bearing exercise to improve lower limb strengthening and balance) was not delivered as intended and the fidelity of the intervention was poor.

### Objective 6. To explore the facilitators’ perceptions of delivering CogEx

The facilitators described participants getting more familiar with the programme over time and could see changes in the participants’ engagement and ability to stand; they understood that standing was important but were not comfortable encouraging people to stand. One facilitator would have preferred a session of only standing exercises. The large number of residents that attended the sessions also contributed to the facilitators not encouraging more people to stand. The sessions should have had no more than eight participants; however, due to the RAC inclusiveness ethos residents that were not research participants took part. One of the facilitators described 11 people attending one session; with only two staff to supervise that many people balancing exercises may not have been safe.

### Objective 7. To explore the participants’ experience of CogEx

Participant engagement was measured by their choice to attend and satisfaction with content [59]. Class attendance was higher for CST than CogEx, and participant focus groups revealed mixed thoughts about the exercises.

While exercises that are challenging can be experienced as hard, that is not an ideal starting point. With a more sedentary population such as that in RAC, starting gradually and building confidence and strength over time may be a better approach. What is not known from the study is how the exercises were presented to the participants, i.e. what the facilitators told them or how they encouraged them. The attendance rates were much higher for CST than CogEx. This could be due to participants not liking the exercises although very few standing exercises were performed, or it could be due to the time of day of the session or the room size as suggested by the RAC2 facility manager.

### Limitations

There were several limitations to this study. While this study sought to upskill and then utilise a healthcare workforce already in place in RAC, there was no additional funding to pay for the programme delivery. The RACs allowed the research to occur with groups that were planned and as such the RACs retained control of the groups. Both RACs had an inclusion policy so if a resident wanted to take part in an offered activity they could. This resulted in residents who were not research participants taking part in both the CST and CogEx groups, and in most cases, these residents had not been eligible to participate in the research due to being wheelchair-bound, hard of hearing or having a MoCA < 10/30. The non-participant residents’ physical capabilities or lack thereof contributed to the facilitators not delivering CogEx as intended (in standing) as they delivered the exercises to the lowest level of physical ability of the group (in sitting).

The outcome measures used were also a limitation. The study initially aimed to test the feasibility of CogEx in community-dwelling and RAC populations and evaluate the appropriateness of the secondary outcome measures. Only one of the outcome measures (Brief BESTest) had normative values for older adults living in RAC, and this poses a challenge for researchers to be able to be confident that changes can be reliably measured and interpreted in this population. The small sample size was too small for inferential analysis or statistical modelling to be undertaken, and so appropriate secondary outcome measures that can be used in the analysis of a larger study remain unknown. However, we found it was reasonably practical to collect a battery of secondary outcome measures in this feasibility study.

A key challenge was the culture of the facilitators to err on the side of caution and not be able to modify the exercise for each individual so that everyone could participate albeit with a variation of the same exercise.

## Conclusion

It was not feasible to deliver CogEx (fall prevention exercises embedded in CST) in the way originally conceived for this trial with the workforce currently delivering CST in Aotearoa New Zealand. The RAC environment is complex and while the CogEx programme appears simple implementation was not. Based on the findings from this study, future research needs to firstly explore either modifying the CogEx training package to give the facilitators more support to develop their skills throughout the 7 weeks of the programme or using a different workforce (e.g. physiotherapist) to deliver the fall prevention component of CogEx with a CST facilitator. Importantly, a greater understanding of the complexity of the RAC setting is needed. Each RAC facility is driven by organisational level factors such as organisational priorities, culture, staffing and workflow pressures but must also deliver on obligations to funders and expectations of resident’s and their families; all of which combine to make each RAC facility a unique environment. Identification and consideration of these factors are needed for successful intervention implementation.

## Supplementary information

**Additional file 1.** The TIDieR (Template for Intervention Description and Replication) Checklist.

**Additional file 2.** CogEx exercises and the muscles and physiological systems targeted.

**Additional file 3.** Recording sheets of level of exercise completed in class (circle or tick).

**Additional file 4.** CogEx participants’ change on each outcome measure.

**Additional file 5.** CST participants’ change on each outcome measure.

## Data Availability

The manual and de-identified datasets analysed during the current study are available from the corresponding author on reasonable request.
