# Supplementary material for: Combining cognitive stimulation therapy and fall prevention exercise (CogEx) in older adults with mild to moderate dementia: a feasibility randomised controlled trial
Source: Pilot Feasibility Stud. 2020 Jul 25;6:108. doi: 10.1186/s40814-020-00646-6 (PMC7382095; doi:10.1186/s40814-020-00646-6)
Supplement: Supplementary file 2 — Additional file 2. CogEx exercises and the muscles and physiological systems targeted. [file 40814_2020_646_MOESM2_ESM.docx]

**Table** CogEx exercises and the muscles and physiological systems targeted

| Exercise | Muscle/group targeted | Physiological effect |
| --- | --- | --- |
| Sit to stand | Quadriceps/Hamstrings/  Gluteals | Strengthening |
| Sideways walking | Hip Abductors/Adductors | Strengthening/balance |
| Calf raises | Gastrocnemius/Tibialis posterior/soleus | Strengthening/balance |
| Standing with feet together |  | Balance |
| Standing heel to bottom | Hamstrings | Strengthening/balance |
| Head  Nod up/down  Turn side/side | Neck extensors/flexors/rotators | Cervical ROM/Vestibular |
| Focus eyes on own fingertip  Move head from side to side  Move head up/down | Eye stabilisers/Neck extensors/flexors/rotators | Vestibular ocular reflex |
| Look at own finger pointing to corner of the room then diagonally to point at the floor |  | Vestibular ocular reflex |
| Elbow to opposite knee | Trunk rotators | Spinal rotation ROM/Vestibular |
| Bend to touch the ground  (all in sitting) | Back extensors | Spinal flexion ROM/Vestibular |
| Backwards chair bends | Truck flexors | Spinal extension ROM/Vestibular |
| Pass object to neighbour  Clockwise  anticlockwise | Trunk rotators | Spinal rotation ROM/Vestibular/Balance |
| Write name on ground with toe |  | Balance |

ROM = range of movement
