# Supplementary material for: Combining cognitive stimulation therapy and fall prevention exercise (CogEx) in older adults with mild to moderate dementia: a feasibility randomised controlled trial
Source: Pilot Feasibility Stud. 2020 Jul 25;6:108. doi: 10.1186/s40814-020-00646-6 (PMC7382095; doi:10.1186/s40814-020-00646-6)
Supplement: Supplementary file 3 — Additional file 3. Recording sheets of level of exercise completed in class (circle or tick). [file 40814_2020_646_MOESM3_ESM.docx]

**Recording sheets** of level of exercise completed in class (circle or tick)

**Session 1 Date:________________________**

| 30s of each exercise | Sit to stand | Use both hands to push up  Push up with one hand  No hand support |
| --- | --- | --- |
|  | Sideways walking | Support  No support |
|  | Calf raises | Support  No support |
|  | Standing with feet together | Support  No support |
|  | Standing heel to bottom | Support  No support |
| Head  Nod up/down  Turn side/side | Sitting  Standing – support   - no support | 10x each in time to the music. Rotate thru movements for 30s |
| Focus eyes on own fingertip  Move head from side to side  Move head up/down | Sitting  Standing– support   - no support | As above |
| Look at own finger pointing to corner of the room then diagonally to point at the floor | Sitting  Standing – support   - no support | Big slow movements for 30s |
| Elbow to opposite knee | Sitting  Standing – support   - No support | 30s |
| Bend to touch the ground  (all in sitting) | To your toes  To the one side then the other  To the opposite foot | 10x each movement rotate thru movements for 30s |
| Backwards chair bends  (sitting) | Sitting arms crossed over chest Standing arms crossed over chest | Sit up tall as you lift your arms, when arms can go no higher gently lean back |
| Pass object to neighbour  Clockwise  anticlockwise | Sitting  Standing – support   - no support | 15s one way 15s the other way |
| Write name on ground with toe | Sitting  Standing – support   - no support | 30s |
| 30s of each exercise | Sit to stand | Use both hands to push up  Push up with one hand  No hand support |
|  | Sideways walking | Support  No support |
|  | Calf raises | Support  No support |
|  | Standing with feet together | Support  No support |
|  | Standing heel to bottom | Support  No support |
