# Supplementary material for: Combining cognitive stimulation therapy and fall prevention exercise (CogEx) in older adults with mild to moderate dementia: a feasibility randomised controlled trial
Source: Pilot Feasibility Stud. 2020 Jul 25;6:108. doi: 10.1186/s40814-020-00646-6 (PMC7382095; doi:10.1186/s40814-020-00646-6)
Supplement: Supplementary file 5 — Additional file 5. CST participants’ change on each outcome measure. [file 40814_2020_646_MOESM5_ESM.docx]

The tables below demonstrate each CST participants’ (n = 10) change on each outcome measure. The change scores have been manipulated such that a positive value represents an improvement on a measure while a negative change represents a poorer performance on a measure (in order to be easily visualised across measures). Participant 4 did not complete a reassessment as they were emotionally unwell, could not complete the MOCA and the assessment was stopped.

**Table 1** CST participants change: MOCA

**Table 2** CST participants change: GDS

**Table 3** CST participants change: QoL: AD

**Table 4** CST participants change: ADAS-Cog 11

**Table 5** CST participants change: Brief BESTest

**Table 6** CST participants change: SPPB

MoCA = Montreal Cognitive Assessment; GDS-15 = Geriatric Depression Scale – 15; QoL: AD = Quality of Life: Alzheimer’s Disease; ADAS-Cog 11 – Alzheimer’s Disease Assessment Scale – Cognitive; Brief BESTest = Brief Balance Evaluation Systems Test; SPPB = Short Form Physical Performance Battery.
